# Perstraction of Intracellular Pigments through Submerged Fermentation of *Talaromyces* spp. in a Surfactant Rich Media: A Novel Approach for Enhanced Pigment Recovery

**DOI:** 10.3390/jof3030033

**Published:** 2017-06-27

**Authors:** Lourdes Morales-Oyervides, Jorge Oliveira, Maria Sousa-Gallagher, Alejandro Méndez-Zavala, Julio Cesar Montañez

**Affiliations:** 1 School of Engineering, University College Cork, Cork, Ireland; lourdesmorales@uadec.edu.mx (L.M.-O.); j.oliveira@ucc.ie (J.O.); M.deSousaGallagher@ucc.ie (M.S.-G.); 2 Department of Chemical Engineering, Universidad Autónoma de Coahuila, Saltillo 25280, Mexico; alejandro.mendez@uadec.edu.mx

**Keywords:** fungal pigments, perstraction, surfactant, *Talaromyces*

## Abstract

A high percentage of the pigments produced by *Talaromyces* spp. remains inside the cell, which could lead to a high product concentration inhibition. To overcome this issue an extractive fermentation process, perstraction, was suggested, which involves the extraction of the intracellular products out of the cell by using a two-phase system during the fermentation. The present work studied the effect of various surfactants on secretion of intracellular pigments produced by *Talaromyces* spp. in submerged fermentation. Surfactants used were: non-ionic surfactants (Tween 80, Span 20 and Triton X-100) and a polyethylene glycerol polymer 8000, at different concentrations (5, 20, 35 g/L). The highest extracellular pigment yield (16 OD_500nm_) was reached using Triton X-100 (35 g/L), which was 44% higher than the control (no surfactant added). The effect of addition time of the selected surfactant was further studied. The highest extracellular pigment concentration (22 OD_500nm_) was achieved when the surfactant was added at 120 h of fermentation. Kinetics of extracellular and intracellular pigments were examined. Total pigment at the end of the fermentation using Triton X-100 was 27.7% higher than the control, confirming that the use of surfactants partially alleviated the product inhibition during the pigment production culture.

## 1. Introduction

In recent years, consumer trends have been oriented to natural products, especially in the food industry. Consumer expectations have led the food processing industry to improve processes in order to deliver high quality-products [1]. To meet the requirements for safer food and also satisfy consumer preferences, scientists are searching for new food additives and new food processing methods [2]. The application of food additives will depend on their function in foods and can be classified as preservatives, nutritional additives, flavouring agents, texturising agents and colouring agents [3]. Natural colourants are obtained from sources like plants [4,5], insects [6] and microorganisms [7]. However, application of microbial pigments still represents a major challenge to biotechnology due to lower extraction or production yields. Dufosse et al. (2014) [8] emphasised in their fungal pigments review, the crucial role that filamentous fungi are currently playing as microbial cell factories, mainly due to the attractive range of stable bio-colourants that they are able to synthesise under controlled conditions. The most-well documented pigment producer fungi is *Monascus* [9]. However, in the last years, pigment producing fungi from other species have gained more attention due to the mycotoxin (citrinin) produced by *Monascus* [10].

Fungi belonging to the genus *Talaromyces* (formerly *Penicillium*) have attracted the attention of scientists due to their high pigment production yields [11,12] and moreover, due to the high thermal stability [13], antioxidant properties [14], antibacterial properties [15] and the absence of toxicity of these pigments [15,16].

However, their successful industrial application will not only depend on their safety or added value properties. The economics of the process need to be assessed early in the design stage before going through more expensive research, such as the scale-up of the process or the characterisation of the molecules. There are various strategies to achieve a cost-effective process such as using cheaper substrates to reduce raw material costs, optimising process conditions to achieve high yields or implementing an efficient product recovery. From these, developing an efficient downstream processing seems to be a rapid strategy to maximise product recovery and to reduce costs.

Pigments produced by *Talaromyces* are excreted out of the cell. However, most remain inside the cell, adding several unit operations to the downstream processing such as cell disruption, extraction of intracellular products, followed by the cell debris removal. Furthermore, a high intracellular pigment concentration could lead to product concentration inhibition. To overcome this issue researchers have been applying an extractive fermentation process, “perstraction”, where the intracellular products are transported out of the cell by using a two-phase system during the fermentation, where the fermentation media represents one of the phases and the other should be an extractive solution. This process has been used in microalgae cultures [17,18] and is also known as “milking”. Ziolkowska and Simon, (2014) [17] reported that “milking algae” is a new successful technology that allows for the reduction of process costs by the extraction of intracellular products, increasing yields and reduction of unit operations. Two aspects are of significant importance for the selection of a suitable extractant for the perstraction of an intracellular product [19]: the biocompatibility of the solvent with the microorganism allowing cell growth, and that the extractant should induce permeability of the cell wall. Recent studies have reported the application of non-ionic surfactant as an effective extractant on the perstraction of intracellular pigments produced by the fungus *Monascus* [20,21]. Wang et al. (2013) [22] reported that the use of non-ionic surfactant Triton X-100 highly increased the pigment production and the extracellular/intracellular pigment ratio by *Monascus* in submerged fermentation. A specific characteristic of the non-ionic aqueous solution is the formation of the cloud point system. The addition of non-ionic surfactant to an aqueous solution forms micelles and when this solution is above the surfactant cloud point, the micelle aqueous solution separates into two phases, a surfactant rich phase (coacervate phase) and a dilute phase [23]. The application of the cloud point system has been reviewed as an advantageous pre-concentration step prior to purification in many bioprocesses, such as extraction of fatty acids from microalgae cultures [18]; extractive fermentation of proteins produced by bacteria [24]; production of l-phenylacetylcarbinol by *Sacharomyces cerevisiae* [25] and in exporting intracellular pigments produced by *Monascus* into its fermentation broth [20,22,26]. 

The application of this concept to submerged fermentation of *Talaromyces* spp. has not been studied, and therefore, the objective of this work was to analyse the effect of various surfactants, at different concentrations, on secreting the intracellular pigments produced by *Talaromyces* spp. into the fermentation broth. The relevance of the precise time of addition of the surfactant was further studied in order to define conditions for maximum extracellular pigments recovery. In addition, the kinetics of pigment production, biomass and substrate consumption during *Talaromyces* spp. culture using non-ionic surfactant micelle aqueous solution were analysed.

## 2. Materials and Methods

### 2.1. Microorganism

*Talaromyces* spp. was used for the production of red pigments (DIA-UAdeC). The purified strain had been previously isolated and characterised as *Penicillium purpurogenum* GH2 [27,28]. *Penicillium purpurogenum* has, however, been transferred to *Talaromyces* spp. [29]. The strain was maintained on PDA (Potato dextrose agar) slants at 4 °C and sub-cultured periodically.

### 2.2. Culture Media

The PDA medium was prepared with a concentration of 39.0 g/L (Bioxon, Mexico). The medium Potato Dextrose Broth (PDB medium, ATCC medium: 336) was prepared by boiling 0.3 kg of finely diced potatoes in 500 mL of water until thoroughly cooked; then the potatoes were filtered through cheesecloth and water was added to the filtrate to complete a volume of 1.0 L. Finally, 20.0 g of glucose is added before sterilisation. The Czapek-dox modified medium [30] consisted in (g/L): d-xylose 15.0, NaNO_3_ 3.0, MgSO_4_·7H_2_O 0.5, FeSO_4_·7H_2_O 0.1, K_2_HPO_4_ 1.0, KCl 1.0 and ethanol 20.0.

### 2.3. Inoculum Preparation

PDB medium was used for the inoculum preparation. Flasks (125 mL) containing 25 mL of PDB medium were sterilised and then inoculated with a spore suspension (1 × 10^5^ spores/mL) of *Talaromyces* spp. previously incubated for 5 days. The flasks were then incubated at 30 °C for 84 h in an orbital shaker (Innova 94, New Brunswick Scientific, Edison, NJ, USA) at 200 rpm [11,31].

### 2.4. Cultivation Conditions

Czapek-dox medium was used for pigment production. The initial pH of the Czapek-dox modified medium was adjusted to 5 before sterilising by using 0.22 m sterile membranes (Millipore, Billerica, MA, USA).

Four different surfactants were tested, assessing their effect on the pigment production and growth in submerged fermentation of *Talaromyces* spp. The surfactants analysed were Tween 80, Span 20, Triton X-100 and polyethylene glycerol polymer PEG 8000, at 3 different concentrations (5, 20, 35 g/L). Surfactants were added at the beginning of fermentation. Extractive fermentation was carried out in 125 mL Erlenmeyer flasks containing 25 mL of medium. Flasks were inoculated with a mycelial suspension 10% (*v*/*v*). The inoculated flasks were incubated at 30 °C in an orbital shaker (Innova 94, New Brunswick Scientific, Edison, NJ, USA) at 200 rpm for 8 days. Control experiments were also performed with no surfactant added.

### 2.5. Analytical Methods

Extracellular pigment recovery was performed according to the methodology reported by Méndez-Zavala et al. (2011) [32]. Each sample was centrifuged at 8000 rpm for 20 min at 4 °C (Sorvall, Primo R Biofuge Centrifugation Thermo, Waltham, MA, USA). The supernatant was then filtered through a 0.45 µm cellulose filter (Millipore, USA). Recovered mycelia were rinsed with distilled water until the supernatant was cleared and used for further extraction of intracellular pigments [20]. Mycelia were soaked in 25 mL of a 70% (*v*/*v*) ethanol aqueous solution which represented the initial fermentation broth volume. Extraction was carried out on an orbital shaker (Inova 94, New Brunswick Scientific, USA) at 200 rpm at 30 °C for 1 h. The concentration of red pigments (extracellular and intracellular) was quantified indirectly by simply measuring the optical density at 500 nm using a spectrophotometer (Cary 50, UV-Visible Varian, Palo Alto, CA, USA). The biomass concentration was determined using the gravimetric method. The analysis of substrate consumption was determined by quantifying the total sugar content using the method reported by [33]. The experiments were replicated three times.

### 2.6. Cloud Point Extraction

Extracellular pigment was separated into a two-phase cloud point system: a surfactant-rich phase and a dilute phase [18]. Pigments-surfactant solution was heated until the formation of these two phases (70 °C) and until reaching the equilibrium (30 min).

An estimation of the pigments that remained in each phase (diluted and coacervate) was done by performing a spectral analysis within the visible wave range (Cary 50, UV-Visible Varian). Various authors have expressed fungal pigments concentration as optical density units at the absorption maxima for a specific wavelength: 400–420 (yellow), 450–470 (orange) and red (490–510) [12,20,26,34,35].

### 2.7. Kinetic Parameters

Pigment extraction productivity (*P_Y/t_*, OD/h), biomass productivity (*P_B/t_*, g/L/h) and yield of product per unit of biomass (*Y_Y/B_*, OD·L/h) were determined by the following equations, [36]:

(1) PYt=Y∞−Yot∞−to

(2) PBt=B∞−Bot∞−to

(3) YYB=Y∞−YoB∞−Bo

where *Y_o_* and *B_o_* correspond to the yield of intracellular pigment (OD_500nm_) and biomass (g/L) at the time (*t_o_*, min) when the surfactant (Triton X-100) was added; *Y_∞_* and *B_∞_* are the yield of pigments (OD_500nm_) and biomass (g/L) at the end of the fermentation (*t_∞_*, min).

### 2.8. Data Analysis

Kinetic parameters were set from experimental data. Statistical analyses were made with Statistica 7.0 software (StatSoft, Tulsa, OK, USA).

## 3. Results and Discussion

### 3.1. Effect of Surfactants on Secretion of Intracellular Pigments into Fermentation Broth (Screening)

**Figure 1:**
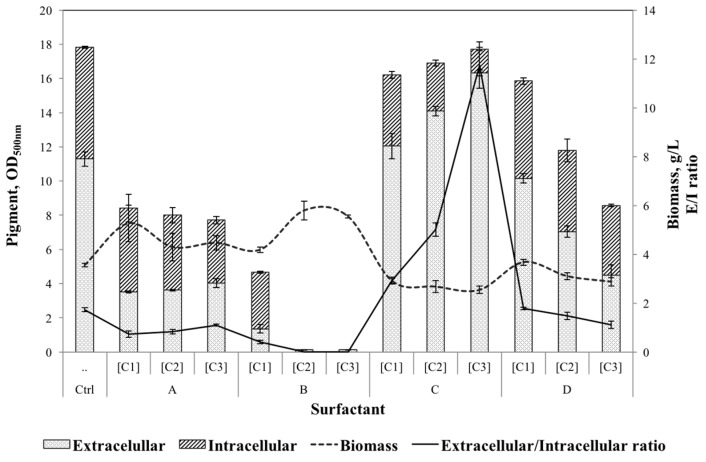
Screening of surfactants’ effect on extracellular and intracellular pigment yield, extracellular/intracellular pigment ratio and growth. Ctrl) Without surfactant, (**A**) polyethylene glycerol polymer PEG 8000; (**B**) Span 20; (**C**) Triton X-100 and (**D**) Tween 80. [Ci, g/L]: C1, 5; C2, 20; C3, 35.

As a first step towards developing a non-ionic surfactant micelle aqueous solution as a fermentation media, a screening of the non-ionic surfactants most commonly used in bioprocess was performed [22,23]. Selection of a suitable surfactant was based on the highest pigment production and extracellular/intracellular pigment ratio. Figure 1 shows the extracellular and intracellular pigments obtained after eight days of fermentation for each surfactant, evaluated at three different concentrations (5, 20 and 35 g/L).

Results for the control media indicated that a high extracellular pigment yield was reached and nearly 40% of the total pigment was still remaining inside of the cell. *Talaromyces* spp. was capable of growing in all surfactants studied; however, pigment production was strongly affected by the addition of surfactant at the beginning of the fermentation. The addition of PEG 8000 caused a reduction in pigment production by more than 50%, in comparison with the control media. This finding limits the use of PEG as a water soluble polymer in aqueous two-phase systems (ATPS) on in-situ product removal processes [37]. When Span 20 was added, the total pigment level was reduced by 80%, in comparison with control media. It was also observed that mycelia morphology changed (dispersed mycelia) and led to a higher biomass growth. Similar behaviour (higher cell growth) was reported [38] on β-Carotene production by *Blakeslea trispora* (Mating type, + and −) with the addition of Span 20. Biomass increment may be due to the change in morphology, i.e., fragmented mycelium led to a major aeration, conditions that allowed higher growth, but were not optimal for pigment production. The addition of Tween 80 at low concentration (5 g/L) caused a reduction of only less than 10% of the total pigment, however, the extracellular/intracellular pigment ratio was increased from 1.72 (control) to 1.79. This may be an indication of the permeability induced on the cell wall by Tween 80; however, increasing the levels of this surfactant resulted in a reduction in both biomass and total pigment, showing the low biocompatibility of this surfactant with *Talaromyces* spp. Similarly, Zhang et al. (2013) [39] reported that the addition of a high concentration of Tween 80 during the production of Antrodin C in submerged fermentation by *A. camphorata* led to massive damage on the cell membrane. When Triton X-100 surfactant was added (35 g/L), an enhancement of extracellular pigment production level (44%) was observed. Total pigment production (extracellular and intracellular) was not statistically different from the control experiments (without addition of surfactant); however, extracellular/intracellular pigment ratio was increased from 1.73 (control) to 11.75. Biomass reduction with elevated concentration of Triton X-100 may indicate that this non-ionic surfactant has a low biocompatibility with the strain, although it may also be related to the secretion of the intracellular pigments. A cell with an elevated concentration of pigment presents a higher weight than an “empty cell”. Triton X-100 has been successfully applied on the secretion of intracellular pigments of *Monascus* [20,26,40], similarly, Triton X-100 has presented high biocompatibility with microbial cell allowing modification of membrane to induce permeability of certain desired metabolites [21,41]. Based on the above results, Triton X-100 (3.5%) was selected for subsequent studies. 

### 3.2. Effect of Addition Time of Triton X-100 during the Fermentation

**Figure 2:**
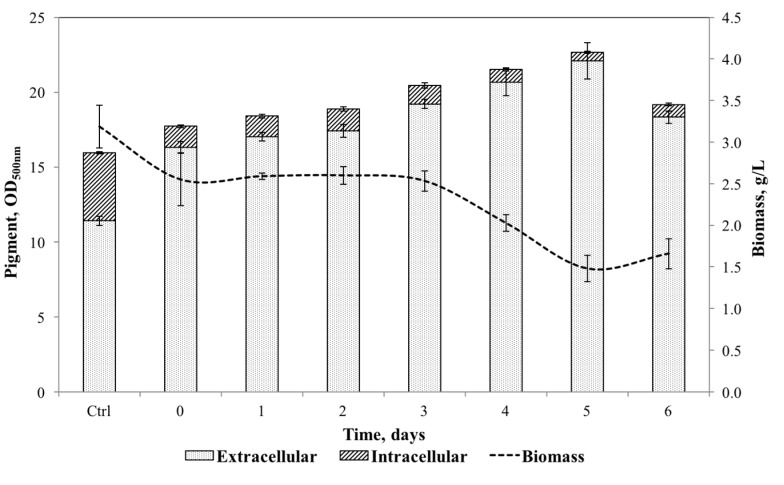
Effect of the addition time of Triton X-100 (35 g/L) on extracellular and intracellular pigment yield and biomass growth.

In the previous experiments, surfactants were added at the beginning of the fermentation. Many authors have added surfactants at different stages of the fermentation to enhance microbial production [39,41]; however, the best time for surfactant addition will vary depending on the microorganism. In order to fully comprehend the effect of Triton X-100 on biomass growth and to enhance pigments production by *Talaromyces* spp., 35 g/L was added at different fermentation times (0, 24, 48, 72, 96, 120 and 144 h). Results for final biomass, extracellular and intracellular pigments are presented in Figure 2. 

Biomass was reduced by 20% when Triton X-100 was added in the interval of 0–3 days and decayed to 50% when it was added at later stages of the fermentation, in comparison with the control. Wang et al. (2013) [22] reported that the addition of Triton X-100 to pigments production by *Monascus* at later stages of the fermentation process resulted in higher biomass growth. In contrast, in this study a consequent addition time of Triton X-100 did not represent better conditions to biomass growth; thus, the results obtained were not sufficient to demonstrate a noticeable Triton X-100 inhibitory effect on the cell. A time course study for each addition time could give more understanding of the Triton X-10 harmful effect on the cell growth, i.e., extended lag phase, reduced maximum biomass and reduced growth rate. However, the main objective of this study was to enhance pigment production and to increase extracellular/intracellular pigment ratio. Figure 2 shows that the excretion of intracellular pigments was considerably enhanced with the addition of Triton X-100; extracellular pigment increased gradually with the time of addition reaching the maximum extracellular pigment (22.1 ± 1.2 OD_500nm_) when Triton X-100 was added at 120 h of fermentation, which corresponds to an increment of 90%, as compared to the control. Furthermore, extracellular/intracellular pigment ratio increased from 2.5 ± 0.1 to 37.9 ± 5.2 for control and Triton X-100 addition at 120 h of fermentation process, respectively. These results are in agreement with previous studies reported on *Monascus pigments* [20,26,40], where it was determined that adding Triton X-100 at a late stage of the fermentation process stimulated intracellular pigment excretion. Based on the above results, the addition of Triton X-100 at 120 h of fermentation at a concentration of 35 g/L was selected for further studies.

### 3.3. Pigments Production Kinetics of Talaromyces spp. by Using Triton X-100

**Figure 3:**
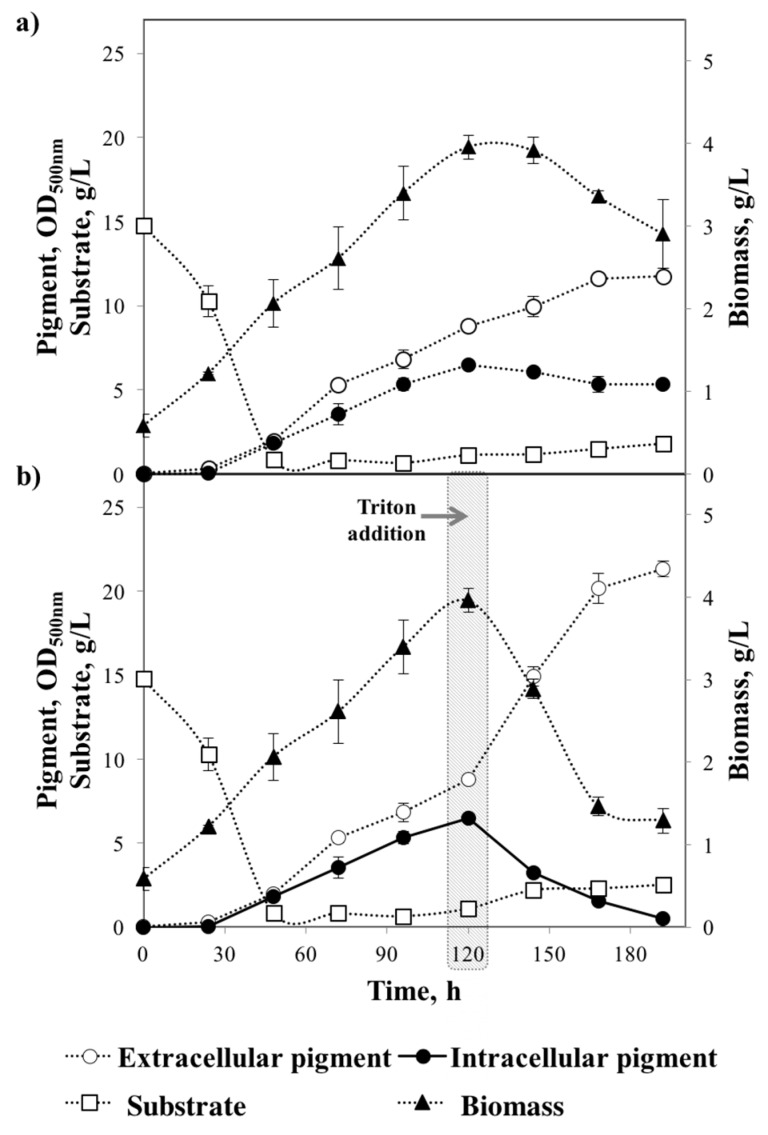
Kinetics of extracellular and intracellular pigment production, substrate consumption and biomass growth. (**a**) Without surfactant; (**b**) Triton X-100 added at 120 h of fermentation.

Kinetics of biomass growth, pigment production and substrate consumption with and without the addition of Triton X-100 at 120 h of fermentation are depicted in Figure 3 (a and b respectively). Figure 3a shows that there is no lag phase for biomass growth; the microorganism started to grow (exponential phase) from 0 to 120 h, then reached a short stationary phase (120 to 150 h), after which biomass weight decayed. There was a slight relation between growth and pigment production. 

Production of pigments started at 24 h and increased gradually until the end of fermentation. It is interesting to note that there is a correlation between extracellular, intracellular pigments and biomass decay. Extracellular and intracellular pigment profiles incremented in a similar pattern until 120 h. After 120 h of submerged fermentation, intracellular pigments decreased correspondingly with the biomass decay and further increment of the extracellular pigments. This behaviour led us to hypothesise that when the fungus reaches the stationary phase (120 h), it stops producing pigments and the further increase of extracellular pigments is partially due to the excretion of intracellular pigments out the cell. This excretion of intracellular pigments may contribute to the biomass decay after 120 of fermentation.

At 120 h of fermentation process, the fungus had reached the stationary phase and thus the addition of Triton X-100 did not cause biomass growth inhibition; this was the reason for the highest extracellular yields being obtained when Triton X-100 was added at this stage. The above results suggested that the surfactant only induced cell permeability and allowed intracellular pigments excretion. Previous work on excretion of *Monascus pigments* have suggested that the addition of Triton X-100 increases the permeability and fluidity of the cell due to the surfactant effect on cell membrane lipids [22]. Furthermore, Deive et al. (2009) [42] reported that the addition of surfactants increased the lipids solubility on the cell membrane changing the cell mass transfer.

The major effect on the cell of the addition of Triton X-100 to the fermentation process could be reflected in the elevated increment of extracellular pigments. Extracellular pigments rose from 8.8 ± 0.2 to 14.9 ± 0.56 OD_500nm_ after 24 h of Triton X-100 addition, which showed this increment as being 5.3 times higher than the control. Furthermore, the extracellular/intracellular pigment ratio was highly increased after 24 h Triton X-100 addition, in comparison with the control, from 1.6 to 4.6. A remarkable observation is that after Triton X-100 addition (120 h), the correlation between extracellular pigments accumulation, intracellular pigments decrease and biomass decay was similar in comparison to the control. This suggests that when the fungus has reached the stationary phase, extracellular pigment increment is only due to the release of intracellular pigments contributing to the higher reduction in biomass weight observed when using Triton X-100 than with the control.

In order to correlate the extraction of intracellular pigments to biomass decay; the pigment extraction productivity (P_Y/t_, OD/h) and biomass productivity (P_B/t_, g/L/h) was obtained after the addition of Triton X-100 for both kinetics (control and perstraction processes). Table 1 shows the parameters obtained. P_Y/t_ and P_B/t_ were 400% higher when using Triton X-100 in comparison with control (negative signs indicate intracellular pigments reduction and biomass decay).

The yield of intracellular product released per unit of biomass was calculated at 120 h of fermentation (time of Triton addition) for both kinetics. Statistical analysis showed that there were no significant differences (95% confidence level) between the Y_Y/B_ obtained in the control and after the addition of Triton X-100. In both cases the cell was releasing approximately 1.90 OD_500nm_ per gram of biomass per litre.

Biomass decay (cell death) has represented a critical concept in microbiology and its estimation is continuously approached by different kinetic models [43]. Van Bodegom (2007) [44] mentioned that there are some inconsistencies related to cell death, because its quantification does not distinguish between biomass losses related to cell lysis or to the actual transport phenomena of intracellular products outside of the cell. In order to understand the mechanism of pigments release and biomass decay relationship, more studies need to be done, due to the variety of components present in the cell. 

**Figure 4:**
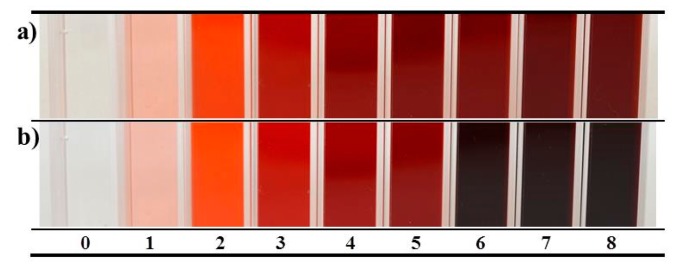
Visual kinetics of colour enhancement. X-axis in days. (**a**) Without surfactant; (**b**) Triton X-100 added at 120 h of fermentation.

Regarding substrate consumption, there was no effect when Triton X-100 was added, as the addition occurred after substrate depletion. At the end of the fermentation process (192 h), extracellular pigments were enhanced above 80% with the addition of Triton X-100 at the end of the exponential growth phase (120 h). Visual kinetics for extracellular pigments enhancement are illustrated in Figure 4. Similar results were obtained by Wang et al. (2013) [22] who reported an increment of 88.4% of pigments produced by *Monascus* by adding Triton X-100 at a late stage of the submerged fermentation. Furthermore, total pigment (extracellular pigment plus intracellular pigment) at the end of the fermentation was 27.7% higher with Triton X-100 compared to control.

### 3.4. Pigments Partitioning in Cloud Point System

**Figure 5:**
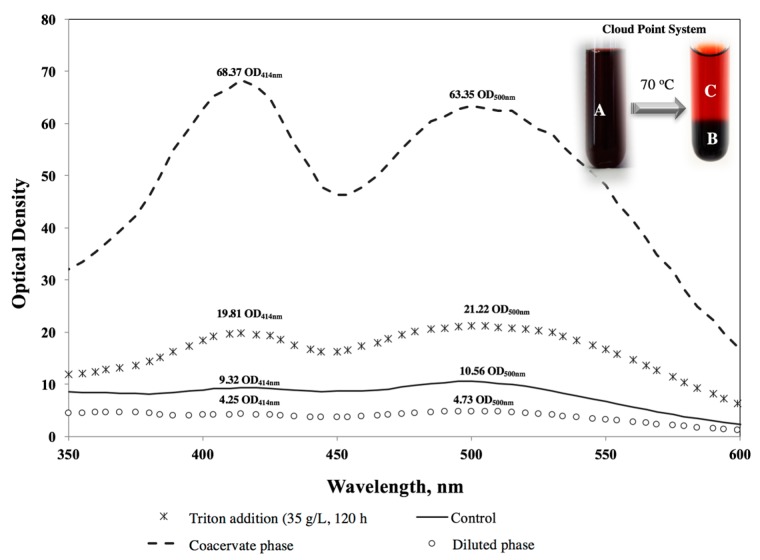
Spectral analysis within the visible wave range performed on the extracellular pigments obtained. Inner Figure: (**A**) the addition of Triton X-100 (35 g/L) at 120 h of fermentation, (**B**) coacervate phase, (**C**) diluted phase.

The inner figure in Figure 5 shows the phase separation of the micelle aqueous solution when it reached a temperature above its cloud point (70 °C). It can be seen that the extracellular pigments obtained were separated between the dilute and coacervate phase. 

Spectral analysis (Figure 5) shows a maximum absorbance at 414 and 500 nm for all the analysed samples. According to previous studies for *Talaromyces* pigments [12,34,45] these peaks correspond to yellow and red, respectively. Optical density values for each peak are also shown in Figure 5.

Red pigments concentration was much higher in the coacervate phase than in the diluted phase (13 times higher). Yellow pigments showed more affinity with the coacervate phase than red pigments, as its concentration was 16 times higher in the coacervate than in the diluted phase. The concentration factor, defined as the ratio of the pigment concentration in the coacervate phase to the concentration in the micelle aqueous solution [24], was 2.98 and 3.45 for red and yellow pigments, respectively. Hu et al. (2012) [20] confirmed the same affinity to the coacervate phase of pigments produced by *Monascus* in a cloud point system by Thin-layer chromatography (TLC) analysis.

## 4. Conclusions

In this study, applying a biphasic fermentation system was shown as a novel and effective bioprocess strategy for an in-situ extraction of intracellular pigments during fermentation, increasing the overall production. Among the studied surfactants, nonionic surfactant Triton X-100 was the most suitable for an in-situ extraction of *Talaromyces* pigments. Under the optimal perstraction conditions, the maximum extracellular pigment yield was 80% higher than the control. Total pigment enhancement (27.7%) confirmed that the addition of Triton X-100 had at least partially alleviated the product inhibition.

Maximising product recovery was evident, and it was demonstrated that the application of the cloud point system could be an advantageous pre-concentration step prior to purification of the pigments produced by *Talaromyces* spp. However, more studies are needed at large scale aired-bioreactors to assess if foaming issues must be addressed.

Also, a techno-economic analysis of the process using perstraction is required to verify if the extraction of pigments during the fermentation reduces, in fact, the costs related to the downstream processing. In any case, using a surfactant rich media can substitute the use of organic solvents for the cell disruption and extraction processes during the downstream processing.

## Figures and Tables

**Table 1:** Intracellular realising (K_I_), biomass decay (K_B_) rates from 120 h to 168 h of fermentation kinetics and K_I_/ K_B_ ratio. (**A**) Control, without surfactant; (**B**) Addition of Triton X-100.

Kinetics	Rate, k		Ratio
	Intracellular, OD_500_/h	Biomass, g/L/h	K_I_/K_B_, OD_500_, L/g
A	−0.023 ± 0.001	−0.0125 ± 0.002	1.90 ± 0.19
B	−0.103 ± 0.004	−0.052 ± 0.004	1.97 ± 0.10
